# What is the place for philosophy within the field of medicine? A review of contemporary issues in medical ethics

**DOI:** 10.1186/s13010-018-0070-3

**Published:** 2018-11-09

**Authors:** Richard Fenton

**Affiliations:** Royal Gwent Hospital, Newport, NP20 2UB UK

## Abstract

This extended essay seeks to unpack some of the key aspects of philosophy which are applicable to medical thought and practice. It proceeds via an analytical discussion of the contemporary debate in three key areas of medical ethics: euthanasia, concepts of health & disease and psychiatry.

The main claims are as follows:

1. The case for legalising euthanasia is strong on philosophical grounds but there are numerous practical obstacles.
2. Elements from the normative and naturalistic definitions of disease are necessary for a thorough definition that dodges common objections to either.
3. Mental health cannot be subsumed under a purely physicalist model of health rendering it distinctly different from other fields in Medicine.

Through a detailed discussion of three salient issues in the philosophy of medicine, it is argued that the application of profound philosophical thought to medicine and its practices reveals a depth that necessitates exploration before simply following the aims of curing all. Philosophical rigour matched with modern medicine has the potential to engage patients and help them make independent, informed decisions and assist physicians to think more clearly, analytically and empathetically.

## Background

This extended essay seeks to unpack the key aspects of philosophy which are applicable to medical thought and practice. Philosophy can broadly be defined as ‘the criticism of assumptions’ [1] in the quest to clarify thought and solidify the foundations of knowledge. In the Western tradition this is typically achieved by an orderly dissection of the individual assumptions inherent to a particular body of knowledge; highlighting these assumptions, subjecting them to relevant objections and examining the validity of counter-examples makes up the bulk of most philosophical discussion. Thus, Philosophy within Medicine has far-reaching applications spanning such topics as subjective wellbeing, bioethics, scientific method and psychiatric illness to name but a few. The essay will proceed in three sections:

1. Euthanasia: The most immediate field for fruitful philosophical thought within Medicine, and the one most frequently encountered by medical students,^1^ is the vast subject of Bioethics. Commonly, a scenario is recounted or devised which places dearly held ethical principles in conflict, demanding a resolution. Since an individual’s intuition alone is insufficient to rationally prefer one course of action over another, some bioethicists invoke various principles, moral theories and empirical evidence to bolster their assertions. Others [2] go as far as to claim that any attempt to establish a universal Bioethics is ultimately futile because morality is relative and therefore the correct course of action is dependent upon local culture. The current key issues championed by bioethicists include voluntary euthanasia, abortion, transplantation and doping; since it is an exemplar of the application of philosophy to medicine and is the subject of current and intense public debate [3], this essay will explore euthanasia in particular.
2. Health and Disease: Modern medicine operates at the unique interface between individual humans and cutting edge science, simultaneously necessitating both a patient centred and an evidence based approach. It has been claimed [4] that the questions of philosophy in medicine are derivative of those of philosophy in other areas, but this ignores the essential psychosocial dimension of medical practice. Medicine is not purely the administration of scientific knowledge upon a physical object – the patient’s body - but the co-ordinated weaving of personal and scientific approaches in which the patient and physician are “co-participants in defining the goal and achieving that goal – cure of illness or promotion of health” [5]. But what is meant by ‘health’ is not always easily discernible. Definitions of ‘health’, ‘disease’ and the demarcation between them shape research agendas and clinical practice. Therefore, a careful examination of these central concepts is essential to any thorough investigation into the nature of medicine.
3. Psychiatry: Over any other medical speciality, psychiatry poses unique problems to an integrated understanding of medicine. The widely referenced Diagnostic and Statistical Manual of Mental Disorders [6] treats mental disorders as clusters of symptoms, yet psychiatric patients are understood to be suffering from mental illness in the same way that an orthopaedic patient suffers from physical illness - the former a disease of the brain, the latter a disease of the bones. This “medical model” [7] seems bolstered by the emergence of dramatically effective biological therapies such as electric convulsive therapy, lithium and tricyclic antidepressants, yet the implementation of talking therapies seems to suppose that mental entities such as beliefs, emotions and perceptions have an independent causal impact. The apparent indispensability of phenomenology^2^ within the diagnosis and treatment of psychiatric illness, and Medicine’s steadfast adherence to a biological model of disease whereby symptoms are necessarily caused by bodily states, poses a deep philosophical conundrum.

### Euthanasia

Central to the euthanasia debate, and indeed much of bioethics, are the 4 ethical principles: beneficence, non-maleficence, justice and autonomy. Since unpacking the meta-ethical underpinnings for these values would be arduous, they are commonly taken to be prima facie principles [8]. It is reasonable to suggest that prohibiting active euthanasia impinges on a patient’s autonomy, denying them the means to end their own life, but this compromise is justified by the principle of non-maleficence whereby killing a patient would constitute harming them under any circumstances. In addition, The Hippocratic Oath prohibits giving “a deadly drug to anybody” a deviation from which could be claimed to compromise trust in physicians and damage the moral centre of medicine [9]. The GMC states that “life prolonging treatment can lawfully be omitted or withdrawn from a patient” [10] but *actively* assisting a suicide is illegal in the UK at present. The contemporary euthanasia debate centres on the distinction between acts and omissions - between allowing someone to die by not acting and someone dying via a physician’s direct act.

Supporters of euthanasia commonly argue [11] that there is no clear moral distinction between acts and omissions. Allowing a child to drown in shallow water by an adult in full knowledge that they are dying could be considered to be as morally reprehensible as the adult who pushes the child into water themselves. Both the act and the omission results in the child’s avoidable death and the adult can be said to be equally blameworthy in both scenarios. Thus, the distinction between acts and omissions is illusory and one of the following options results:

1. *Both* active and passive euthanasia should be permitted on the basis that in certain dire and consensual circumstances a patient’s death would be a good thing.
2. *Both* active and passive euthanasia should be prohibited on the basis that they are equally tantamount to killing the patient.

Dissolving the distinction between acts and omissions places active and passive euthanasia on equal footings as they both involve intentionally ending a patient’s life. Since modern Medicine makes it possible (but not always desirable) to keep human beings biologically alive for a long time, one’s conviction in the physician’s legitimacy to withdraw treatment under adverse circumstances in a patient’s best interests commits one to also permit active euthanasia. This argument highlights that if active euthanasia is wrong, it cannot be simply on the basis that it is an act rather than an omission. Additional grounds for the moral distinction between active and passive euthanasia are discussed below.

Another basis for the illegality of active euthanasia might be that additional negative consequences could result. Firstly, it must be considered that permitting active euthanasia might weaken society’s commitments to provide optimal care for dying patients; as euthanasia would undoubtedly offer a cheaper alternative to invasive medical intervention, society might become less committed to providing expensive technological and pharmacological support to dying patients, and even more so at times of pressure on funding. However, this objection is based on a claim with little evidential support, and suggesting that offering active euthanasia will erode the quality of care of dying patients equates to suggesting that recognising a patient’s right to forego life-sustaining treatment will have the same effect. However, there is no persuasive evidence to that end despite withdrawal of treatment being regularly implemented. Therefore, this debate is essentially an empirical disagreement about the impact of legalising active euthanasia, but without a clear specification of how a policy would be enacted and how the potential for abuse would be diminished, these imagined consequences are largely speculative.

Secondly, it could be claimed that permitting voluntary active euthanasia may inadvertently open the door to instances of involuntary active euthanasia. It is not always easy to accurately assess whether an individual’s choice is unequivocally voluntary, such as in cases of coercion, and this element of doubt may be sufficient to prohibit active euthanasia altogether. In addition to inadvertent cases of involuntary euthanasia, there could be reason to expect that the legalisation of active euthanasia would result in pressure to legalise it in incompetent patients who are unable to express their own wishes, in much the same way that life-sustaining treatment is regularly withdrawn in the best interests of patients lacking capacity. This objection points to the fact that the consequential legalisation of the non-voluntary form of active euthanasia could greatly increase the numbers of patients on whom it might be performed, amplifying the potential for misapplication and abuse yet further.

Finally, issue is taken with the legalisation of euthanasia on the grounds of legal intrusion. The present situation whereby collaborating physicians form decisions to forego life-sustaining treatment does not require regular intervention by the judicial system. Due to the emotive nature of the issue, the introduction of lethal injections or overdoses may blur the line between medical and legal decisions, particularly in light of the issues with establishing the freedom of patient decision-making already discussed. This could also potentially undermine the hard battles medical experts have fought in order to retain autonomy from the judicial system when making decisions to withdraw treatment. While it is plausible that procedures could be designed to clearly establish the voluntariness of the patient’s request without invoking legal support, it would be naïve to suppose that this would be acceptable to the majority of society in the first instance.

Thus the case for the legalisation of euthanasia is strong on philosophical grounds but the practical concerns raised by the introduction of active euthanasia into a hitherto unfamiliar medical system presents an obstacle. It is the responsibility of advocates of active euthanasia to demonstrate clearly workable public policy which would effectively regulate and respectfully scrutinise the practice.

### Health and disease

It is uncontroversial to claim that many of the activities of Medicine aim to improve health. However, the concepts of ‘health’ and ‘disease’ are not clearly defined; questions about the normality of ageing and the exact cut-off for hypertension pierce the comfortable complacency surrounding these notions. In addition, the precise meaning of ‘health’ may change according to gender, age and culture. The World Health Organisation (WHO) makes the broad statement that ‘health is a state of complete physical, mental and social well-being and not merely the absence of disease or infirmity’ [12]. This is a useful and inclusive definition but it does not guide us when determining whether a certain condition is to be classified as ‘a disease’. Reznek [13] highlights the significance of such classifications in virtue of the impact it has on society and politics:

“The classification of a condition as a disease carries many important consequences. We inform medical scientists that they should try to discover a cure for the condition. We inform benefactors that they should support such research. We direct medical care towards the condition, making it appropriate to treat the condition by medical means such as drug therapy, surgery, and so on. We inform our courts that it is inappropriate to hold people responsible for the manifestations of the condition. We set up early warning detection services aimed at detecting the condition in its early stages when it is still amenable to successful treatment. We serve notice to health insurance companies and national health services that they are liable to pay for the treatment of such a condition. Classifying a condition as a disease is no idle matter [13].”

There are two main ideologies in the debate surrounding disease classification: normative and naturalistic. A normative conception of disease categorises certain conditions in virtue of their undesirability for the patient. However, categorising a condition as a disease should a patient consider the condition bad for them leads us to consider ugliness, shortness and hunger, for example, as pathological. In addition, some conditions which we would intuitively call diseases may confer some survival benefit upon us, such as when infestation with certain parasitic worms dampens unwanted immune responses [14] or cowpox confers immunity to smallpox. A more complex normative approach identifies disease with anything internal which involuntarily causes at least one of the three ‘3 Ds’ – disability, discomfort or deformity [15]. However, this falls foul of the objection that each one of these ‘D’ concepts is subjective and therefore a definition inclusive of these concepts will itself be subjective by extension.

A naturalistic view categorises disease according to a natural conception of biological dysfunction. This approach benefits from being potentially applicable to multiple organisms and from being more amenable to defining objectively. Borse [16] identifies a disease with physiological dysfunction of an organ or process, whereby ‘dysfunction’ is defined as subnormal function “under some arbitrarily chosen minimum level below the mean.” This statistical definition of disease, while leaving open to debate the exact cut-off point for sufficiently deviant physiology, dodges the objection from subjectivity which impeded the normative account. However, it is open to an objection derived from common sense: if every dysfunction sufficiently different from the physiological norm is deemed to be a disease then we would label organ donors and those with unusual immune receptors as ‘diseased’. Thus, it is apparent that elements of the normative and the naturalistic definitions of disease are necessary for a holistic understanding of the concept in order to prevent misapplications of the term ‘disease’. Therefore, a full understanding of the concept ‘disease’ includes both physiological dysfunction and deprivation of the individual in the present or the future.

### Psychiatry

Psychiatry poses a challenge to the Philosophy of Medicine due to the distinctive character of mental illness. Much like physical illness, mental disorders cause pain and distress in a myriad of different guises. As in non-psychiatric Medicine, psychiatric doctors are trained to apply the principles of diagnostic rigour, evidenced based therapies and effective communication skills to provide their patients with optimum care. However, there is less scientific clarity regarding the nature of mental illness than there is regarding most physical diseases, and Psychiatry’s digressions from medical practice as a whole places it in a unique position within the field. This section seeks to clarify elements of the conceptual framework which justifies Psychiatry’s inclusion as a medical specialty and argues in favour of a phenomenological understanding of mental illness.

As discussed in the previous section, in order to delineate a condition as sufficiently divergent from normal functioning to be labelled ‘a disease’ we must have a clear conception of ‘health’. This is challenging in the case of the mind because the psychological norms of different cultures and subcultures vary much more markedly than those of physiological norms. It is tempting to try to define ‘mental abnormality’ as simply ‘psychology that is undesirable to the patient’ but mental illness may not always be apparent to the sufferer; social norms play an integral role in determining whether certain behaviours are manifestations of mental illness and so one’s embeddedness within society is an important factor. In addition, the victims of psychiatric problems are sometimes subject to treatment under Section, against their will, which is seldom the case in non-psychiatric medicine; this is another feature which suggests that an understanding of mental illness from a purely a physiological perspective is too narrow a conception.

Exponents of the medical model consider psychiatry to be an applied branch of the cognitive neurosciences and mental illness a pathological process in the brain which causes the clinically observable symptoms that are elicited by psychiatrists. This position identifies the mind with the brain, conceptualising human beings as purely physical machines of which ‘the mind’ is one component. Whether the mind consists in information processing systems or molecular manifestations of gene expression, a physicalist approach places the mind squarely within the brain of the patient. This approach receives favour as it sits comfortably within our current scientific model, allowing us to use characteristic symptoms of mental illness as a temporary placeholder for the underlying physical nature of the disease and many successes have resulted from it; Alzheimer’s disease for example; a previously poorly understood condition, has now been identified with markers such as cerebral atrophy, amyloid plaques and neurofibrillary tangles [17] deduced from post-mortem evidence. Likewise, the medical model holds that the patho-physiology of conditions such as depression and schizophrenia is similarly available for investigation within the brains of patients, and that a complete and perfectly detailed understanding of the brain processes involved will be wholly explanatory of the condition.

This perspective seems to bypass the essential fact that, unlike with, say, heart disease, there is an indispensable experiential element to mental illness bound up in its definition; we may not sensibly claim that someone is suffering from depression, for example, if they do not experience irrational low mood or suicidal ideation. In addition, simple low mood or suicidal ideation themselves are not sufficient to diagnose someone with depression as there are situations where there may be rational cause for someone to feel low or to consider suicide. Upon unpacking the concept of depression, we can see it presupposes an understanding of the concept of rationality, but unlike the norms of physiology, the norms of rationality are not amenable to scientific investigation. The rationality of a certain thought is determined not by empirical evidence but by culturally established rational principles and thorough inquiry of the patient. Thus behaviour and experience is understood to be functioning well when it is directed towards the achievement of certain understandable human goals, and while it seems platitudinous to say that a brain is required to do that, it seems that there are additional essential yet subjective elements to achieving an understanding of abnormal behaviour and experience.

Thus we can see, mental disorders are in some ways like physical disease and can be understood as deviations from human norms. In the case of mental disorders, to deviate from a norm is to think, feel, intend, act, and so on, in ways which are not found to be readily intelligible by those considered to be normal. This impacts upon one’s ability to interact within one’s community and in this sense, a mental disorder is one which affects the whole of one’s being. While the intellectual traditions, basic concepts and clinical as well as research strategies of Medicine can readily be applied to Psychiatry, there are extra features inherent to the treatment of mental health which cannot be subsumed under a purely physicalistic model.

## Conclusion

Through a detailed discussion of three salient issues in the philosophy of medicine, it has been argued that the application of profound philosophical thought to medicine and its practices reveals a depth that necessitates exploration before simply following the aims of curing all. Philosophical rigour matched with modern medicine has the potential to engage patients and help them make independent, informed decisions and assist physicians to think more clearly, analytically and empathetically.

## Footnotes

1 The graduate will be able to behave according to “ethical and legal principles” - Tomorrow’s Doctors, page 25

2 descriptive psychology conceived in the works of Husserl, Heidegger, Sartre & Merleau-Ponty which, when studying the structure of various types of experience, places the first person perspective at the centre of investigation. The discipline highlights the indispensable ‘intentionality’ of consciousness – the nature of thought as directed towards a certain object in the world; that is to say, thoughts are always ‘about’ something.
